# Reproduction of Mucohaemorrhagic Diarrhea and Colitis Indistinguishable from Swine Dysentery following Experimental Inoculation with “*Brachyspira hampsonii”* Strain 30446

**DOI:** 10.1371/journal.pone.0057146

**Published:** 2013-02-27

**Authors:** Joseph E. Rubin, Matheus O. Costa, Janet E. Hill, Heather E. Kittrell, Champika Fernando, Yanyun Huang, Brendan O’Connor, John C. S. Harding

**Affiliations:** 1 Department of Veterinary Microbiology, Western College of Veterinary Medicine, University of Saskatchewan, Saskatoon, Saskatchewan, Canada; 2 Department of Large Animal Clinical Sciences, Western College of Veterinary Medicine, University of Saskatchewan, Saskatoon, Saskatchewan, Canada; 3 Prairie Diagnostic Services Inc., Saskatoon, Saskatchewan, Canada; Auburn University, United States of America

## Abstract

**Background:**

Mucohaemorrhagic diarrhea caused by *Brachyspira hyodysenteriae,* swine dysentery, is a severe production limiting disease of swine. Recently, pigs in western Canada with clinical signs indistinguishable from swine dysentery were observed. Despite the presence of spirochetes on fecal smears, recognized *Brachyspira* spp. including *B. hyodysenteriae* could not be identified. A phylogenetically distinct *Brachyspira*, called “*B. hampsonii”* strain 30446, however was isolated. The purpose of this study was to experimentally reproduce mucohaemorrhagic colitis and characterize strain 30446 shedding following inoculation.

**Methods and Findings:**

Eighteen 13-week-old pigs were randomly assigned to inoculation (n = 12) or control (n = 6) groups in each of two trials. In trial 1, pigs were inoculated with a tissue homogenate collected from clinically affected field cases. In trial 2, pigs were inoculated with a pure broth culture of strain 30446. In both trials, mucohaemorrhagic diarrhea was significantly more common in inoculated pigs than controls, all of which remained healthy. In animals with mucohaemorrhagic diarrhea, significantly more spirochetes were observed on Gram stained fecal smears, and higher numbers of strain 30446 genome equivalents were detected by quantitative PCR (qPCR). Strain 30446 was cultured from colon and/or feces of all affected but no control animals at necropsy.

**Conclusions:**

*“Brachyspira hampsonii”* strain 30446 causes mucohaemorrhagic diarrhea in pigs following a 4–9 day incubation period. Fecal shedding was detectable by day 4 post inoculation, and rarely preceded the onset of mucoid or haemorrhagic diarrhea by more than 2 days. Culture and 30446-specific qPCR are reliable methods of detection of this organism in feces and tissues of diarrheic pigs. The emergence of a novel *Brachyspira* spp., such as “*B. hampsonii”*, creates diagnostic challenges including higher risk of false negative diagnostic tests. We therefore recommend diagnostic laboratories routinely use *Brachyspira* culture, *nox*-based and species-specific PCR, and DNA sequencing to diagnose *Brachyspira*-associated colitis in pigs.

## Introduction

Swine dysentery is a mucohaemorrhagic colitis causing severe production losses in pigs, resulting from infection with the intestinal spirochaete *Brachyspira hyodysenteriae*. The first clinical description of swine dysentery was published in 1921, although it was not until 1971 that *B. hyodysenteriae* (then *Treponema hyodysenteriae*) was recognized as the cause [1]. Spirochetal colitis is a less severe illness caused by *B. pilosicoli*, which is characterized by diarrhea (non-haemorrhagic with a wet cement consistency) and poor feed conversion in chronic cases [2]. Other organisms within the genus *Brachyspira* are varyingly associated with disease, including *B. murdochii, B. intermedia, B. innocens* and the provisionally named ‘*B. suanatina’*, although considerable strain level differences in pathogenicity (particularly among *B. intermedia*) are apparent [3]–[5]. In October 2009, grow-finish pigs with clinical signs indistinguishable from swine dysentery were observed in a commercial barn in Saskatchewan, Canada [6], [7]. Tissues, carcasses and rectal swabs collected from a number of affected pigs over several months were submitted to Prairie Diagnostic Services Inc. (PDS) at the University of Saskatchewan in Saskatoon, Canada. Fibrinous mucohaemorrhagic colitis and typhlitis with superficial necrosis was observed grossly. Histologically, sub-acute to chronic muco-purulent to fibrino-suppurative colitis with superficial necrosis was observed. No recognized pathogens could be identified. All samples were negative for *Lawsonia intracellularis* and *Salmonella* spp., and despite large numbers of spirochetes seen on Gram strained fecal smears, *B. hyodysenteriae* and *B. pilosicoli* were not detected. The apparent spirochetosis prompted further testing of samples by PCR using genus-specific primers targeting the *Brachyspira* NADH oxidase (*nox*) gene [8]. The sequence of this 939 bp PCR amplicon was identical to clade 2 of the recently described, provisionally named *“Brachyspira hampsonii”* [9]. The particular strain identified in western Canada and used in these trials is named 30446. Although strain 30446 is also phenotypically indistinguishable from *“B. hampsonii*” clade 2 [9], there is distinct variability within *“B. hampsonii”* (clades 1 and 2), and the pathogenicity of strain 30446 may not be reflective of all *“B. hampsonii”* isolates. As the species has not been formally recognized this study will refer precisely to *“B. hampsonii”* strain 30446.

The purpose of this study was to investigate the pathogenicity of *“Brachyspira hampsonii”* strain 30446 in experimentally infected pigs. The results of two infection trials in grower pigs, involving inoculation with either tissue homogenate (trial 1) or pure culture (trial 2) are presented.

## Materials and Methods

### Ethics Statement

Both trials were designed and conducted in accordance with the Canadian Council for Animal Care and approved by the University of Saskatchewan Committee on Animal Care and Supply (Protocol #20110038).

### Trial 1. Tissue Homogenate Inoculation

#### Source of strain 30446

*“Brachyspira hampsonii”* strain 30446 infected material was obtained from clinically affected 13-week-old pigs from a porcine reproductive and respiratory syndrome (PRRS) negative farm. Following necropsy, the colonic and caecal mucosa were removed from the underlying sub-mucosa and muscularis by scraping with the edge of a glass microscope slide, and then frozen at −80°C within four hours of collection. To confirm the absence of pathogens other than strain 30446, sections of small and large intestine were processed routinely for histopathology, bacterial culture, and PCR.

#### *Brachyspira* culture

*Brachyspira* was cultured by streaking out approximately 10 µg of feces or intestinal contents onto BJ and CVS agar plates [10], [11]. Plates were incubated anaerobically using a commercial system (Anaerogen, Oxoid Limited, Basingstoke, United Kingdom) at 42°C for 48 hours. Bacterial colonies were not formed, instead, positive cultures were indicated by zones of strong β-haemolysis from which motile spirochetes could be seen microscopically.

#### DNA extraction and PCR

DNA was extracted from samples using either the QIAmp DNA stool mini kit (feces or colon contents) or DNEasy blood and tissue kit (cultured bacteria or terminal colon tissue) (Qiagen Inc., Toronto, Ontario) and 2 µl of extract used as template in PCR reactions. DNA was extracted in triplicate in terminal colon tissues and colonic contents. To differentiate strain 30446 from other *Brachyspira* spp., partial 16S rRNA, *cpn*60, *nox*, *adh*, *alp*, *est*, *gdh*, *glp*K, *pgm* and *thi* were amplified and sequenced using previously published primers (Table 1).

**Table 1:** Primer sequences used to detect and identify *Brachyspira* spp.

Target Gene	Application	Primer Name	Primer Sequence (5′-3′)	Reference
*Nox*	*Brachyspira* genus specific	NOX F	TGG CAT ACT ATC TCA TCA	[8]
		NOX R	GAT GGA AGC TAT ATG TAT CTT A	
*Adh*	MLST scheme	ADH-F206	GAA GTT TAG TAA AAG ACT TTA AAC C	[12]
		ADH-R757	CTG CTT CAG CAA AAG TTT CAA C	
*Alp*	MLST scheme	ALP-F354	TCC AGA TGA GGC TAT ACT TC	[12]
		ALP-R1262	TAT GCT CTT TTT GCT AAT ATT G	
*Est*	MLST scheme	EST-F229	GAT GCT TCA GGC GGA GTT ATG	[12]
		EST-R847	CCA CAC TCA TAG CAT AAA TAC TG	
*Gdh*	MLST scheme	GDH-F514	GGA GTT GGT GCT AGA GAG AT	[12]
		GDH-R1157	ATC TCT AAA GCA GAA GTA GCA	
*Glpk*	MLST scheme	GLPK-F123	AGG CTG GGT AGA ACA TAA TGC	[12]
		GLPK-R1158	TCT TTA CTT TGA TAA GCA ATA GC	
*Pgm*	MLST scheme	PGM-F172	GTT GGT ACT AAC AGA ATG AAT A	[12]
		PGM-R1220	CCG TCT TTA TCG CGT ACA TT	
*Thi*	MLST scheme	THI-F163	TGT GTT ATA CAA TCA GCA CTT C	[12]
		THI-R1079	GTA GTA AGT ATT CTA GCT CCA G	
*Nox*	*Brachyspira* sp. 30446 SYBR assay	JH224	TCG CTA AAT TAT TCC AAC AAG GA	This study
		JH225	AAA CGC ATT TCT ATT CCA GCA	
*cpn* **60**	*Brachyspira hyodysenteriae* SYBR assay	JH0073	AGT GAA ATA GTT GCT CAT ATC AAA T	This study
		JH0074	GCA TCA CTG ATT AAA GAA CCA AT	
*cpn* **60**	*Brachyspira pilosicoli* SYBR assay	JH0077	ACA ATG ATA AAG AGA TAG GTG CTT	This study
		JH0078	CTA ATG AAA GGC TAG TTT CTA ATG AT	

To specifically detect *“B. hampsonii”* strain 30446, *B. hyodysenteriae* and *B. pilosicoli,* SYBR green qPCR assays were developed targeting either *nox* (strain 30446), or *cpn*60 (*B. hyodysenteriae* and *B. pilosicoli*) (Table 1). Product sizes were 215 bp for strain 30446, 120 bp for *B. hyodysenteriae* and 111 bp for *B. pilosicoli.* Quantitative PCR reactions were conducted on a Bio-Rad MyiQ thermocycler with iQ SYBR green supermix (Bio-Rad Laboratories (Canada) Ltd., Mississauga, Ontario) according to the manufacturer’s instructions. Quantification was accomplished by use of a serially diluted standard curve of plasmids containing target sequences. All reactions were run in duplicate and each run included both extraction negatives and no template controls. For samples that resulted in a C_t_ value higher than the lowest standard, but with dissociation curves consistent with the expected product, a result of detected but not quantifiable (DNQ) was reported. The detection limits of the assays were defined by the linear portion of the standard curve for each assay, which were 10^1^–10^7^ copies per reaction (10^3^–10^9^ copies per gram of feces or tissue) for *B. hyodysenteriae* and *B. pilosicoli*, and 10^2^–10^7^ copies per reaction (10^4^–10^9^ copies per gram of feces or tissue) for *“B. hampsonii”*.

#### Pigs

Eighteen five-week old, Landrace male weanling piglets were purchased from a PRRS negative high health commercial farm in Saskatchewan, Canada, with no history of swine dysentery or previous laboratory diagnosis of *Brachyspira* spp. The pigs were conveniently selected, of average body weight compared to their cohorts and all appeared healthy. Farm selection was based on the screening of four and seven week old pigs from three farms for *B. hyodysenteriae, B. pilosicoli* and strain 30446 prior to trial 1. Animals were randomly assigned to control (CTRL, n = 6) and inoculated (INOC, n = 12) groups on arrival, and held for a 10-day acclimation period prior to inoculation. They were fed a commercially prepared, non-medicated, pelleted starter diet *ad libitum,* and housed in separate rooms in 4′×6′ pens each containing 3 pigs. During the acclimation period all pigs were tested for strain 30446, *B. hyodysenteriae* and *B. pilosicoli* in feces by qPCR ten, seven and five days and immediately prior to first inoculation. Fecal DNA extracts for pre-trial screening were tested in duplicate from DNA extraction through PCR (independent technical replicates were done from DNA extraction through PCR).

#### Preparation of inoculum

**Figure 1:**
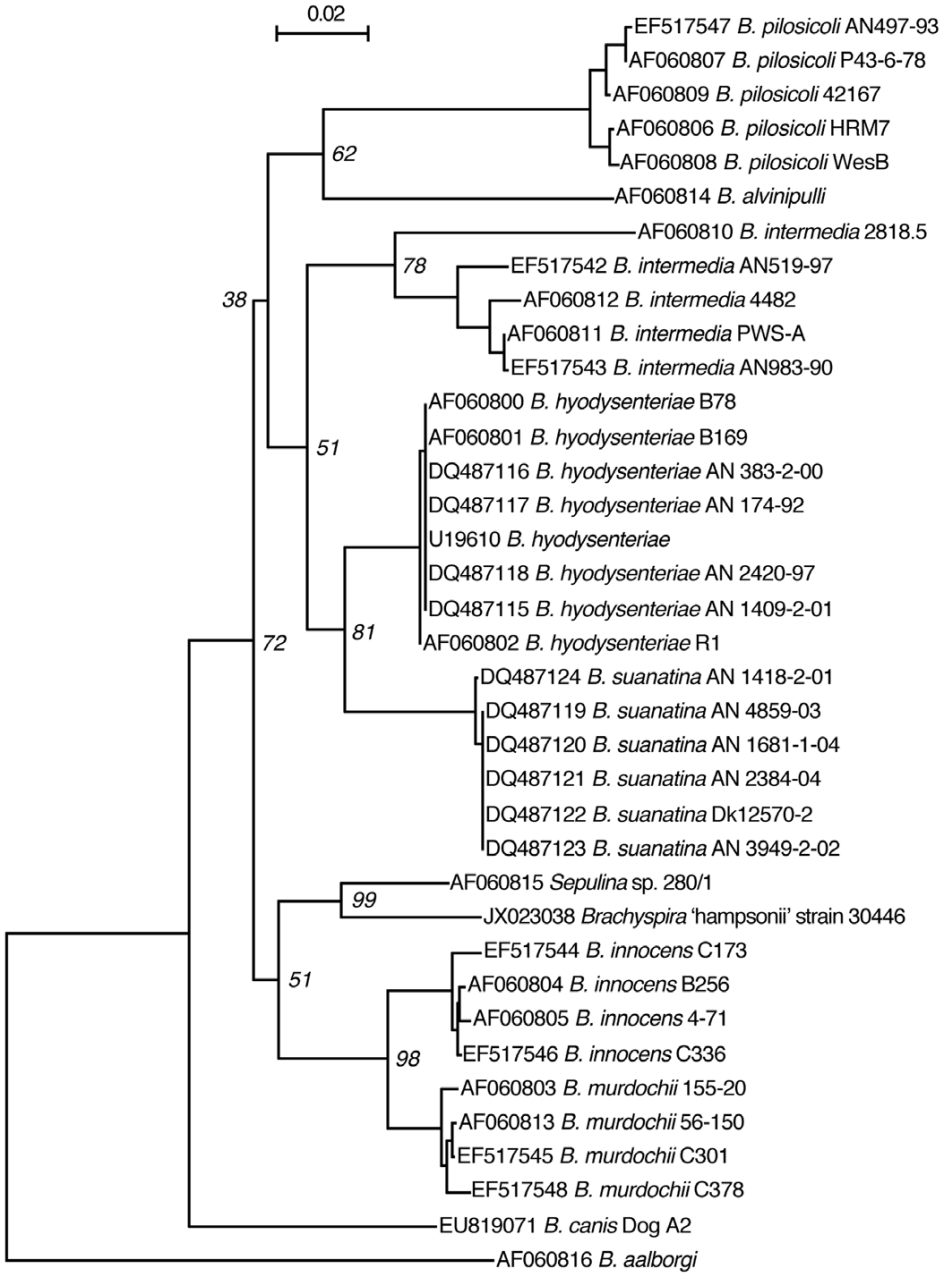
Phylogenetic tree of *Brachyspira* spp. Phylogenetic tree based on alignment of 810 bp of the *nox* gene of *Brachyspira* spp., including *“B. hampsonii”* strain 30446. The alignment was created using CLUSTALw, followed by distance calculation (F84 matrix) and neighbour joining using PHYLIP. The tree is a consensus of 100 boostrap iterations, and bootstrap values are indicated at the major nodes. GenBank accession numbers for *nox* sequences are indicated in the tree. Scale bar indicates 0.02 substitutions per site.

The inoculum was prepared by mixing approximately one part mucosal scraping and three parts 0.1 M pH 7.0 phosphate buffered saline (PBS) in a sterile blender. Strain 30446 was identified in the inocula and differentiated from other *Brachyspira* species by a novel *nox* sequence (Figure 1) and using a previously published MLST protocol for *Brachyspira* spp. [12]. Of the seven published MLST primer pairs, amplicons were not generated from three. Unique sequences were generated from *pgm* (Genbank accession JX469445, 93% sequence identity to *B. murdochii*), *thi* (JX469446, 92% sequence identity to *B. hyodysenteriae*), *glpk* (JX469444, 95% sequence identity to *B. murdochii*) and *est* (JX469443, 93% sequence identity to *B. murdochii*). The concentration of *“B. hampsonii”* strain 30446 in the inoculum was determined by qPCR and three daily inoculum doses of 3.42×10^8^, 1.80×10^8^ and 6.37×10^7^ genome equivalents were given. These doses were intermediate to recent trials with *B. murdochii* where 10^6^ colony forming units were used and a *‘B. suanatina’* sp. nov. trial where 30 ml of a 10^8^ to10^9^ cells/mL inoculum was used [5], [13].

#### Inoculation of pigs

Pigs were inoculated on three consecutive days (D0, D1 and D2) as previously described [13], [14]. To decrease gastric transit time, feed was removed 16 hours prior to, and returned one hour after inoculation. Prior to inoculation, pigs were sedated with 8 mg/kg IM azaperone (Stresnil, Vetoquinol Canada Inc., Lavaltrie, Quebec). Pigs in the INOC group were intra-gastrically inoculated using an 18 French feeding tube, followed by 50 mL of sterile PBS (0.1 M, pH 7.0). CTRL pigs were mock inoculated with 50 mL of PBS.

#### Observation of pigs and daily sample collection

The pigs were observed and scored twice daily for responsiveness, skin colour, appetite and body condition, respiratory effort and fecal consistency. Fecal consistency was scored daily as: 0 = formed, normal; 1 = soft, wet cement consistency; 2 = runny or watery; 3 = mucoid diarrhea; or 4 = bloody diarrhea. Gram stained fecal smears were made from rectal swabs collected daily from each pig. An investigator blinded to the slide ID, evaluated and scored all slides as: 0 = negative; 1 = less than 1 spirochete/high power field (hpf); 2 = between 2 and 10 spirochetes/hpf; 3 = between 11 and 49 spirochetes/hpf; 4 = greater than 50 spirochetes/hpf. Fecal samples collected from each pig on days 3, 7, 10 and 14 post inoculation were also tested by qPCR for *“B. hampsonii”* strain 30446. For statistical analysis, the qPCR results were categorized: 0 = not detected; 1 = DNQ; and 2 = quantifiable.

#### Necropsy

After the intensity of mucohaemorrhagic diarrhea peaked (INOC), or at the end of the study (CTRL) on day 16, pigs were euthanized by cranial captive bolt and exsanguinated. A complete necropsy was performed with special attention to the stomach, duodenum, jejunum, ileum, spiral colon, caecum and rectum. Samples for histological examination, *Salmonella* culture on brilliant green agar following enrichment in selenite broth (pooled colon and caecum), *Lawsonia intracellularis* PCR (ileum) [15], porcine circovirus 2 (PCV2) immunohistochemistry (ileum, mesenteric lymph node) [16] and porcine reproductive and respiratory syndrome virus PCR (serum; Tetracore Inc., Rockville, MD) were submitted to PDS. Colonic tissue and contents were tested by qPCR for *B. hyodysenteriae*, *B. pilosicoli* and *“B. hampsonii”* strain 30446 in triplicate as done in pre-trial screening. To detect viable strain 30446, colonic tissue was cultured for *Brachyspira* and if isolated, was speciated by sequencing *nox* PCR amplicons.

#### Histology

The pathologist (YH) responsible for analysis of the samples was blinded to the identity of the slides. The presence or absence of superficial necrosis and inflammation were scored in Haematoxylin-Eosin stained sections of colon, caecum and rectum. Scoring for inflammation was based on the severity of neutrophil infiltration in the mucosa and fibrinous exudate on the surface. Necrosis of the mucosa was assessed by visualization of apoptotic cells and degenerated nuclei. Additionally, Warthin-Faulkner stained colon sections were examined for the presence of *Brachyspira*-like organisms associated with the lesions.

#### Statistics

Statistical analysis was performed using SPSS version 18.0 (SPSS Inc., Chicago, IL). The presence or absence of mucohaemorrhagic diarrhea in INOC versus CTRL groups was compared using the Fisher’s exact test. The presence or absence of histologic and gross lesions in INOC pigs with or without mucohaemorrhagic diarrhea, and CTRL pigs was compared using the Fisher’s exact test. Spirochete slide score from colonic swabs and strain 30446 DNA concentration (0 = negative, 1 = DNQ, 2 = quantifiable) in INOC pigs with and without mucohaemorrhagic diarrhea, and in CTRL pigs were compared using the Kruskal-Wallis test followed by post-hoc Mann-Whitney test if significant. Two-tailed *P*-values ≤0.05 were considered significant.

### Trial 2. Pure Broth Culture Inoculation

The methodology for trial 2 was similar to that of trial 1; only major differences will be noted below. Pigs were sourced from the same farm following pre-screening of three and six week old pigs for *B. hyodysenteriae, B. pilosicoli* and *“B. hampsonii”* strain 30446. Randomly assigned CTRL (n = 6) and INOC (n = 12) pigs were held for an 8 day acclimation prior to inoculation. Groups were housed in separate rooms containing 2 pigs (INOC) or 3 pigs (CTRL) per pen. During the acclimation period, feces were collected eight, five and two days, and immediately prior to first inoculation and tested for *Brachyspira* spp. by PCR and culture as described above.

*“B. hampsonii”* strain 30446 was cultivated *in vitro* on JBS broth from one of the pig colons used in trial 1 (isolate 6953). Approximately 2 cm^2^ of solid media with haemolytic zones were used to inoculate JBS broth (brain heart infusion with 5% (v/v) fetal calf serum, 5% (v/v) sheep’s blood, and 1% (w/v) glucose). Broth cultures were incubated in glass vials with magnetic stir bars anaerobically at 39°C for 24 hours with constant stirring.

*“B. hampsonii”* strain 30446 was administered by intra-gastric tube for three consecutive days at doses of 2.78×10^6^, 5.04×10^8^ and 4.50×10^8^ genome equivalents, followed by 50 ml PBS. CTRL pigs were mock inoculated with an equivalent volume of sterile JBS broth followed by 50 ml PBS. One INOC pig (#683) did not receive a complete dose of inoculum on D1, and the CTRL group inadvertently did not have feed removed prior to the second inoculation. Daily observations and fecal consistency scoring was performed as described above. The Gram stained smears made from rectal swabs were scored: 0 = less than 1 spirochete/hpf; 1 = between 2 and 10 spirochetes/hpf; 2 = between 11 and 49 spirochetes/hpf; 3 = greater than 50 spirochetes/hpf. Daily rectal swabs were cultured for *Brachyspira* and results were scored: 0 = negative; 1 = less than 10 colonies/1° streak; 2 = less than 10 colonies/2° streak; 3 = less than 10 colonies/3° streak; or 4 = less than 10 colonies/4° streak. Fecal samples collected daily from each pig were tested by qPCR for *“B. hampsonii”* strain 30446. For statistical analysis, the qPCR results were categorized: 0 = not detected; 1 = DNQ; and 2 = quantifiable.

Necropsy examinations were performed after the intensity of mucohaemorrhagic diarrhea peaked (INOC), on day 14 for non-diarrheic INOC, or day 15 for CTRL. Histopathologic and microbiologic assessments of tissues were performed as described above. *Brachyspira* cultured from feces collected on the day of euthanasia and from terminal colon tissues was verified by *nox* PCR and speciated by sequencing.

Statistical analysis was identical to that described for trial 1 above. In addition, daily fecal culture results in INOC pigs with and without mucohaemorrhagic diarrhea, and in CTRL pigs were compared using the Kruskal-Wallis test followed by post-hoc Mann-Whitney test if significant.

## Results

### Source Farm Screening

All pigs tested at the source farm during the pre-trial screening were negative for *B. hyodysenteriae* and *B. pilosicoli* by qPCR. DNQ levels of *“B. hampsonii”* strain 30446 were found in 1/20 pigs prior to trial 1 and 3/20 pigs prior to trial 2. As DNQ levels of strain 30446 were detected at all three potential source farms tested, the farm with the lowest prevalence was used to supply the pigs for both trials. The pre-trial screening was conducted on different pigs from those used in each experiment.

### Trial 1. Tissue Homogenate Inoculation

**Figure 2:**
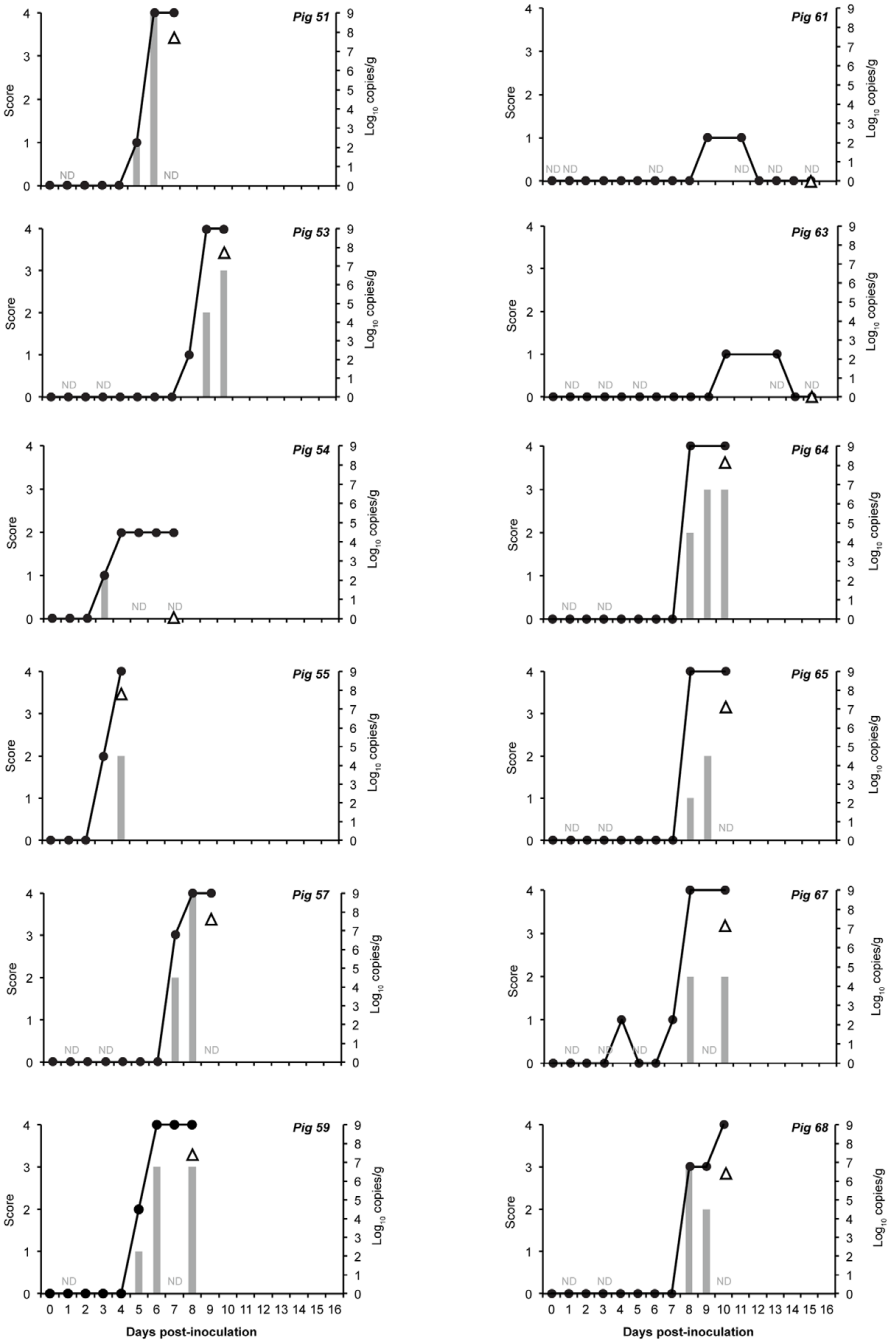
Fecal consistency, shedding and tissue concentrations in pigs following inoculation with tissue homogenate containing *“Brachyspira hampsonii”* strain 30446. Daily fecal consistency scores (line, left ordinate; 0 = formed, normal; 1 = soft, wet cement consistency; 2 = runny or watery; 3 = mucoid diarrhea; or 4 = bloody diarrhea). Fecal smear spirochete scores (grey bars, left ordinate: 0 = negative; 1 = less than 1 spirochete/high power field (hpf); 2 = between 2 and 10 spirochetes/hpf; 3 = between 11 and 49 spirochetes/hpf; 4 = greater than 50 spirochetes/hpf). Strain 30446 DNA concentration (copies/g, triangles, right ordinate) in colon tissue samples collected at necropsy. Pig IDs are indicated in the upper right corner of each panel. ND = fecal smear spirochete score not done.

During the acclimation period, 3/12 INOC pigs had DNQ levels of strain 30446 DNA present in feces at single time points (Table 2). The study continued for 16 days following the first inoculation. All CTRL animals remained healthy throughout the study, and spirochetes were never seen in the feces nor detected by qPCR. In some INOC pigs, strain 30446 was detected in feces at very low levels (DNQ) on day 3 post inoculation (PI) (#53, #54, #55, #65), day 7 PI (#64) and at quantifiable levels on day 7 PI (#64; 2.67×10^5^ genome equivalents/g) and day 10 PI (#68; 4.45×10^7^ genome equivalents). Of the 12 INOC pigs, nine developed mucohaemorrhagic diarrhea between days 4 and 10 PI (Figure 2). Of the remaining three INOC pigs, two (#61 and #63) developed soft feces on days nine and ten, which resolved on days 12 and 14 PI respectively. Pig #54 developed watery diarrhea on day four PI. Neither blood nor mucous were observed from any of these three animals. Mucohaemorrhagic diarrhea was significantly more common in INOC (9 of 12) than CTRL (0 of 6) (*P*<0.001).

**Table 2:** Detection of *“B. hampsonii”* strain 30446 in pre-inoculation screening of fecal samples during the acclimation period^1^.

Trial 1				Trial 2				
Pig ID	Day -10	Day -7	Day -5	Pig ID	Day -8	Day -5	Day -2	Day 0
**Inoculated**				**Inoculated**				
**51**	–	–	–	683	–	–	–	–
**53**	–	–	–	684	–	–	–	6.80×10^4^
**54**	–	–	–	686	–	–	–	–
**55**	–	–	–	688	DNQ	–	–	–
**57**	–	–	–	689	DNQ	–	–	–
**59**	DNQ	–	–	690	–	–	DNQ	DNQ
**61**	–	–	–	693	–	–	–	–
**63**	–	–	–	694	–	–	DNQ	–
**64**	–	–	DNQ	695	–	–	–	DNQ
**65**	–	–	–	696	DNQ	–	DNQ	DNQ
**67**	–	–	DNQ	697	DNQ	–	–	–
**68**	–	–	–	700	DNQ	–	–	DNQ
**Control**				**Control**				
**52**	–	–	–	685	–	–	–	DNQ
**56**	–	–	–	687	DNQ	–	–	–
**58**	–	–	–	691	DNQ	–	–	DNQ
**60**	–	–	–	692	–	–	–	–
**62**	–	–	–	698	–	–	–	–
**66**	–	–	–	699	–	–	–	DNQ

1 Quantitative PCR results for all pigs at ten, seven and five days prior to inoculation in trial 1, and eight, five and two days prior to inoculation, and at inoculation in trial 2. Hyphens indicate that strain 30446 was not detected, DNQ indicates detectable but not quantifiable concentrations, and a number indicates the concentration of strain 30446 in genome equivalents/g of feces.

In pigs that developed mucohaemorrhagic diarrhea, the number of spirochetes seen on Gram stained slides increased concurrently with elevated fecal consistency scores (Figure 2). In one INOC pig (#67), transient mild diarrhea was observed four days before the first spirochetes were seen, and three days prior to the onset of persistent diarrhea (Figure 2). Of the three INOC pigs that did not develop mucohaemorrhagic diarrhea, spirochetes were not observed from two (#61 and #63), transient low level spirochete shedding was observed on a single day in the other (#54) (Figure 2). Significantly higher terminal spirochete slide scores were seen in pigs with mucohaemorrhagic diarrhea than either INOC pigs without mucohaemorrhagic diarrhea (*P* = 0.011) or CTRL (*P*<0.001). Similarly, in terminal colon, strain 30446 concentrations were significantly higher in pigs with mucohaemorrhagic diarrhea (1.84×10^7^–1.32×10^8^) than either INOC without mucohaemorrhagic diarrhea (negative - DNQ, *P* = 0.009) or CTRL (negative, *P*<0.001). Neither *B. hyodysenteriae* nor *B. pilosicoli* were detected in terminal colon tissue or contents from any pig.

*Lawsonia intracellularis, B. hyodysenteriae, B. pilosicoli, Salmonella* spp., and PRRS virus were not detected in terminal samples from any pig. A single INOC pig (#57) was weakly positive for PCV2, and all other animals were negative. Culture of colonic tissue revealed *“B. hampsonii”* strain 30446 in nine, and *B. intermedia* (98% identical over 805 bp of the *nox* gene to *B. intermedia* ATCC 51140^T^) in one (#54) INOC pigs. Two INOC pigs (#61 and #63) were culture negative. The *nox* sequence of an isolate similar to *B. intermedia* (97% identical over 824 bp of the *nox* gene to *B. intermedia* ATCC 51140^T^) was also isolated by culture from one CTRL pig (#56).

Gross pathological findings were consistent with clinical signs. Lesions were significantly more common in pigs with mucohaemorrhagic diarrhea than in INOC pigs without mucohaemorrhagic diarrhea (Table 3). In affected pigs, enlargement of the mesenteric lymph nodes, fibrinous typhlocolitis, meso-colonic edema and/or congestion and abundant mucohaemorrhagic caecal and rectal contents were seen. Small intestinal lesions were seen in four INOC pigs including serosal congestion of the ileum (n = 1) and jejunum (n = 3). Hyperkeratosis of the gastric epithelium was seen in 14 pigs (INOC n = 8 and CTRL n = 6). Mild to moderate erosions were seen in the pars esophagea of 5 pigs, one INOC with mucohaemorrhagic diarrhea, one INOC without mucohaemorrhagic diarrhea, and three CTRL.

**Table 3:** Comparison of histological and gross lesions and spirochete numbers on Warthin-Faulkner stained colonic sections^1^.

Experiment		Histologic Lesions			Gross Lesions			Spirochetes on Silver Stain	
		Colon	Caecum	Rectum	Colon		Caecum	Absent-Rare	Abundant
					Mucoid and/or haemorrhagic colitis	Edema and congestion	Muco-fibrinous typhlitis		
**Trial 1 Tissue Homogenate**	Control	0/6^†^	0/6^†^	0/6^†^	0/6^†^	0/6^†^	0/6^†^	2/6	0/6
	Inoculated	9/12	5/12	6/12	7/12	9/12	7/12	3/12	9/12
	Diarrheic	9/9*^†^	5/9^†^	5/9^†^	7/9*^†^	9/9*^†^	7/9*^†^	0/9	9/9
	No diarrhea	0/3*	0/3	1/3	0/3*	0/3*	0/3*	3/3	0/3
**Trial 2 Pure Culture**	Control	0/6^‡^	0/6^‡^	0/6^‡^	0/6^‡^	0/6^‡^	0/6^‡^	6/6	0/6
	Inoculated	7/12	6/12	8/12	7/12	5/12	5/12	4/12	8/12
	Diarrheic	7/8^#‡^	6/8^‡^	8/8^#‡^	7/8^#‡^	5/8^‡^	5/8^‡^	0/8	8/8
	No diarrhea	0/4^#^	0/4	0/4^#^	0/4^#^	0/4	0/4	4/4	0/4

1 Statistical comparisons were among CTRL (n = 6), INOC with mucohaemorrhagic diarrhea (n = 9) and INOC without mucohaemorrhagic diarrhea (n = 3) in trial 1. In trial 2, comparisons were among CTRL (n = 6), INOC with mucohaemorrhagic diarrhea (n = 8) and INOC without mucohaemorrhagic diarrhea (n = 4) in trial 2.

† ‡ # *Within column, animals with similar superscripts are statistically different (*P*≤0.05).

**Figure 3:**
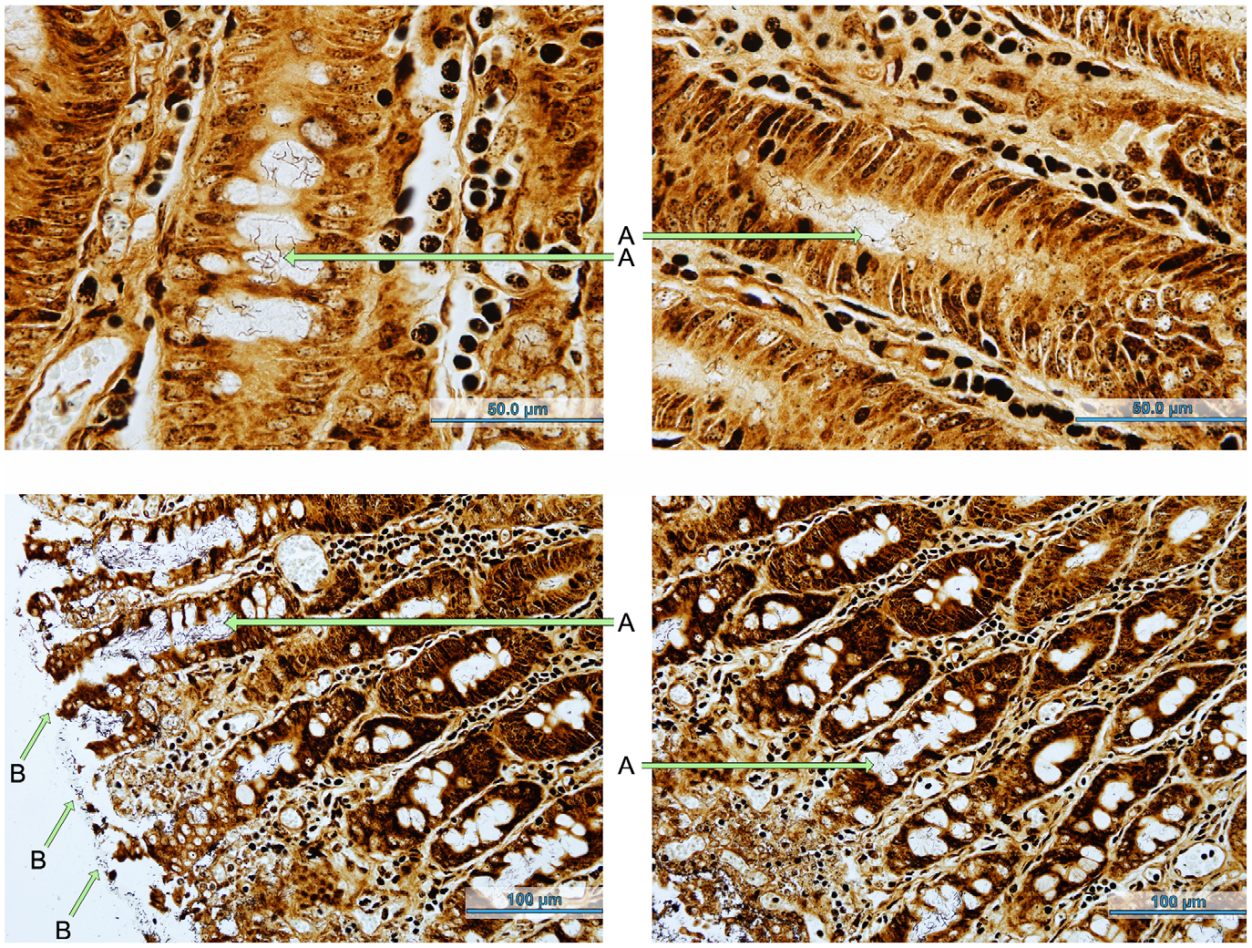
*“Brachyspira hampsonii”* strain 30446 in Warthin-Faulkner stained colonic sections. Histologic sections of spiral colon taken from pigs with mucohaemorrhagic diarrhea examined with Warthin-Faulkner silver staining. Spirochetes can be seen in the intestinal crypts (A) and along the epithelial surface (B).

Histologically, necrosis of the superficial colonic mucosa, which was covered by a bacteria-rich, mucoid exudate that extended into the superficial crypts, was observed. When examined using Warthin-Faulkner silver stains, numerous long, thin spirochetes were seen in the crypts and among the mixed bacteria on the surface (Figure 3). There was mild but variable congestion and haemorrhage in the lamina propria, and a mild neutrophil infiltration in the lamina propria and lumen in some cases. Lesions were most consistent in the colon but were also detected in the caecum and rectum. Lesions were significantly more common in pigs with mucohaemorrhagic diarrhea than in either INOC without mucohaemorrhagic diarrhea or CTRL (Table 3).

### Trial 2. Pure Broth Culture Inoculation

*“B. hampsonii”* strain 30446 was detected at DNQ levels in various INOC (8 of 12) and CTRL (4 of 6) pigs during the acclimation period, but generally as single events (Table 2). One INOC pig (#684) had a quantifiable level of strain 30446 (6.80×10^4^ copies/g) on the day of inoculation.

**Figure 4:**
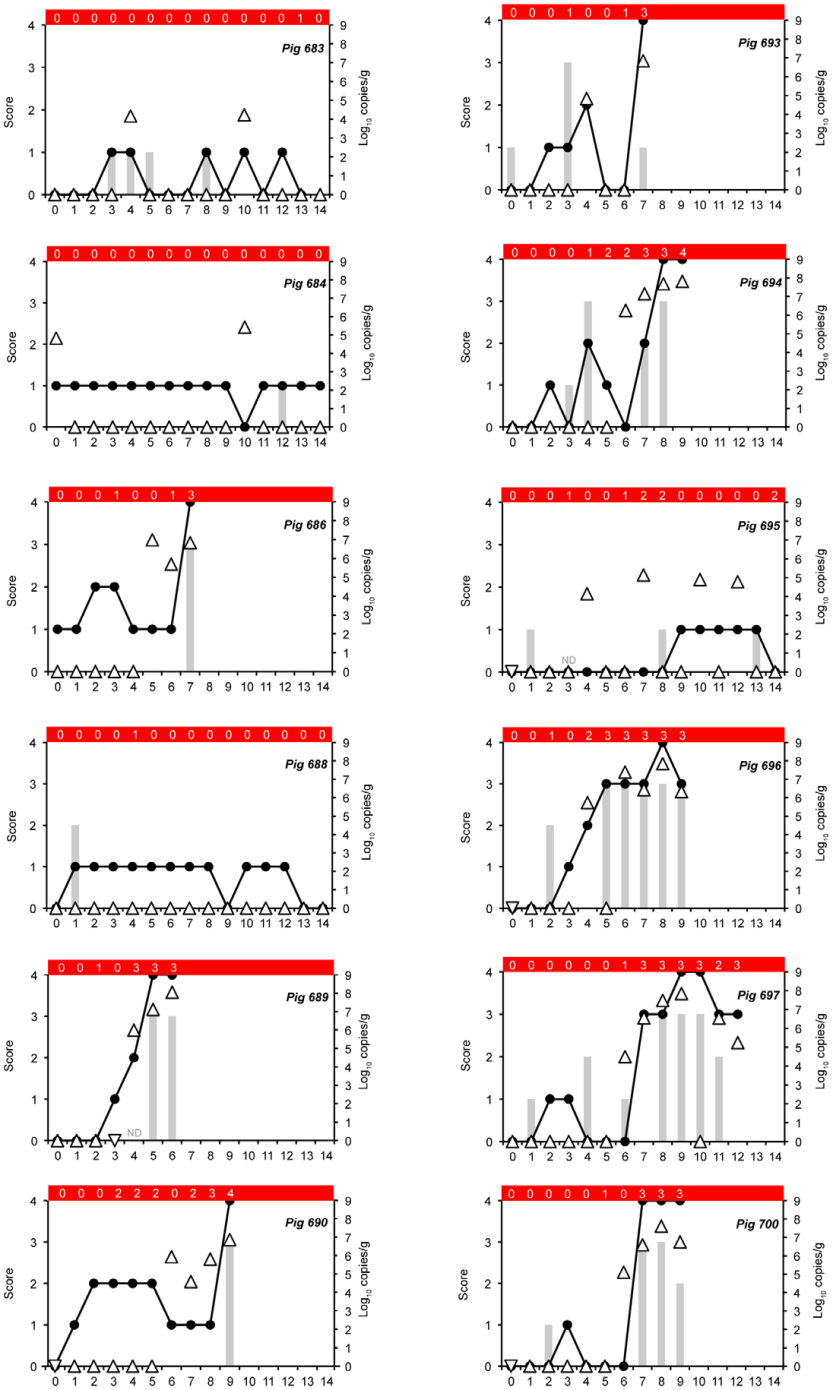
Fecal consistency, shedding and tissue concentrations in pigs following inoculation with pure broth cultivated *“Brachyspira hampsonii”* strain 30446. Fecal consistency scores (line, left ordinate: 0 = formed, normal; 1 = soft, wet cement consistency; 2 = runny or watery; 3 = mucoid diarrhea; or 4 = bloody diarrhea). Fecal smear spirochete scores (grey bars, left ordinate: 0 = less than 1 spirochete/high power field (hpf); 1 = between 2 and 10 spirochetes/hpf; 2 = between 11 and 49 spirochetes/hpf; 3 = greater than 50 spirochetes/hpf). Strain 30446 DNA concentration in feces (triangles, right ordinate), upside down triangles indicate DNQ. The red bar at the top of each panel indicates the semi-quantitative fecal culture score (0 = negative; 1 = less than 10 colonies/1° streak; 2 = less than 10 colonies/2° streak; 3 = less than 10 colonies/3°streak; 4 = less than 10 colonies/4° streak). Pig IDs are indicated in the upper right corner of each panel. ND = fecal smear spirochete score not done.

**Figure 5:**
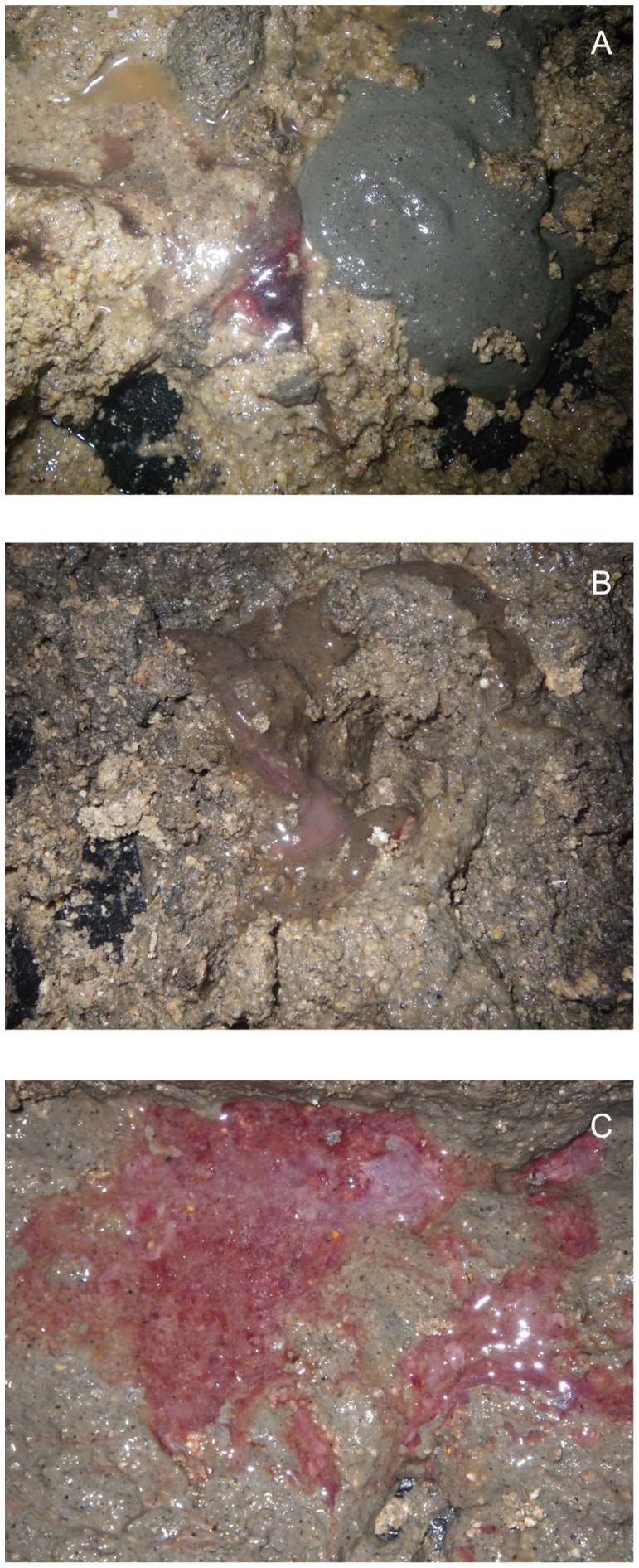
Mucohaemorrhagic diarrhea associated with *“Brachyspira hampsonii”* strain 30446. Fecal consistency following inoculation with pure broth “*Brachyspira hampsonii*” strain 30446 culture ranged from that similar to wet cement (A, right side), clots of blood (A, left side) or mucus (B), or severe watery mucohaemorrhagic diarrhea (C). Images A, B, C were taken on days 5, 6 and 8 PI respectively.

**Figure 6:**
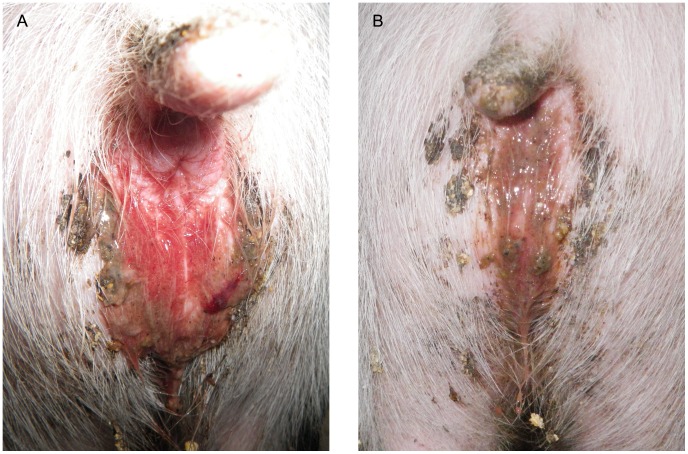
Perianal fecal staining. In some pigs, mucohaemorrhagic feces adheres to the perianal region of pigs following inoculation with pure broth *“Brachyspira hampsonii”* strain 30446. Both images are taken on day 8 PI: frank blood with a clot (A), blood with mucus (B).

The study continued for 14 days following the first inoculation. All CTRL animals remained healthy throughout the study. Although low numbers of spirochetes were seen by direct microscopic examination in feces of CTRL pigs, no sample was positive by qPCR or culture during following inoculation. Of the 12 INOC pigs, eight developed mucohaemorrhagic diarrhea between 5 and 9 days PI (Figures 4, 5, 6). Of the remaining four INOC pigs (#683, #684, #688 and #695) soft feces were variably observed between days 1 and 14. Low levels of strain 30446 shedding (1.48×10^4^ to 2.67×10^5^ genome copies/g) were detected by qPCR in the feces of three of these pigs (#683, #684 and #695), and positive culture results were obtained for #683, #688 and #695. Mucohaemorrhagic diarrhea was significantly more common in INOC (8 of 12) than CTRL (0 of 6) (*P* = 0.013).

While fecal consistency score, spirochete slide score, culture results and qPCR counts varied daily, when elevated, they tended to move together as a group (Figure 4 **)**. Gram stain slide scores of 3+ were seen before or concurrently with diarrhea among pigs that developed mucohaemorrhagic diarrhea, whereas scores above 2+ were not observed in pigs without mucohaemorrhagic diarrhea. Similarly, 2+ or 3+ cultures were only observed in pigs that developed mucohaemorrhagic diarrhea (Figure 4). Compared to CTRL or INOC without mucohaemorrhagic diarrhea, INOC pigs with mucohaemorrhagic diarrhea had significantly higher terminal spirochete slide scores (*P* = 0.009 for INOC with diarrhea, *P = *0.026 for INOC without diarrhea) and more frequent isolation of 30446 by culture (*P* = 0.001 for INOC with diarrhea, *P = *0.004 for INOC without diarrhea). A significantly higher number of genome equivalents of strain 30446 DNA was detected by qPCR in the colonic tissue of pigs with mucohaemorrhagic diarrhea than CTRL (*P*<0.001). Although the same association was seen between pigs with mucohaemorrhagic diarrhea (9.65×10^3^–1.85×10^7^ genome copies/g) and INOC without mucohaemorrhagic diarrhea (DNQ - 1.03×10^4^ genome copies/g), the non-parametric method of analysis was insensitive to this difference (*P = *0.157). Sequencing of *nox* PCR amplicons from terminal fecal and colon tissue cultures revealed isolates with 99–100% sequence similarity to strain 30446 in all pigs with mucohaemorrhagic diarrhea and one INOC pig without mucohaemorrhagic diarrhea (#684). An isolate with sequence similarity to *B. intermedia* (97% identity over 825 bp of the *nox* gene to *B. intermedia* ATCC 51140^T^) was grown from one other INOC pig without mucohaemorrhagic diarrhea (#695). All CTRL pigs were culture negative. Importantly, *nox* sequence of the *Brachyspira* species isolated from the terminal fecal samples from the INOC group matched the *nox* sequences of the isolates from terminal colon culture.

*Lawsonia intracellularis, B. hyodysenteriae, B. pilosicoli, Salmonella* spp. and PRRS were not detected in terminal samples from any pig. Pig #695 tested positive for PCV2 in ileum and lymph node by immunohistochemistry, while all others tested negative.

#### Necropsy

**Figure 7:**
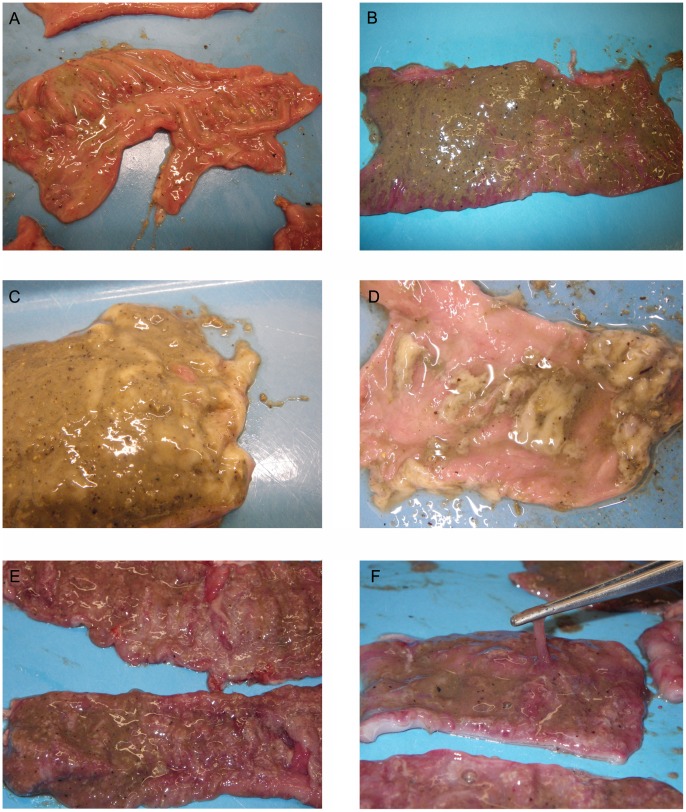
Colonic mucosal lesions associated with *“Brachyspira hampsonii”* strain 30446. Gross colonic mucosal lesions observed following inoculation with pure broth culture containing strain 30446. Images are of different INOC pigs euthanized between D6 and D12 post-inoculation. A = mild to moderate hyperemia and/or congestion with scant mucous deposited on mucosal surface; B = moderate to severe mucosal congestion with normal looking contents adhering to mucosa; C = moderate to severe fibrinomucoid exudate adhering to hyperemic mucosal surface prior to washing; D = patchy fibrinomucoid exudate adhering to hyperemic mucosal surface after washing; E = severe fibrinonecrotic colitis; F = thick adherent mucoid exudate on mucosal surface of colon.

Gross pathological findings were consistent with the clinical signs. Lesions were significantly more common in pigs with mucohaemorrhagic diarrhea than in INOC pigs without mucohaemorrhagic diarrhea or CTRL (Table 3). In affected pigs, lesions were typified by mucoid or mucohaemorrhagic colitis and fibrino-mucoid typhlitis. Lesions ranging in severity from mild congestion to severe fibrino-necrotic colitis with profuse mucous were seen (Figure 7). Small intestinal lesions were found in three pigs with mucohaemorrhagic diarrhea and one INOC without mucohaemorrhagic diarrhea, and consisted of mild corrugation and thickening of the ileum grossly. Hyperkeratosis of the pars esophagea was seen in 14 pigs (INOC n = 8, CTRL n = 6). Additionally, one pig with mucohaemorrhagic diarrhea had a well demarcated area of superficial necrosis in the gastric fundus, and one INOC pig without mucohaemorrhagic diarrhea had small erosions of the pars esophagea.

The distribution of histologic lesions was significantly associated with clinical signs and was similar to that observed in trial 1 (Table 3). There were no abnormal findings in either CTRL or INOC without mucohaemorrhagic diarrhea. Inflammation and/or necrosis of the superficial mucosa of the colon, caecum and rectum were seen in all affected pigs.

Amplicons were generated from *nox* PCR from terminal colon tissue from all INOC pigs. Sequencing revealed 11 amplicons with 99–100% sequence identity to strain 30446 (including all affected pigs) and one amplicon with 97% sequence identity to *B. intermedia* ATCC 51140^T^ (from #695, an INOC pig that did not develop mucohaemorrhagic diarrhea). When tested by qPCR, strain 30446 specific amplicons were generated for all 12 INOC pigs and no CTRL pigs. When sequenced, 10 of these amplicons were 99–100% similar to strain 30446, while sequencing failed for the other two. The colon contents from one CTRL pig (#685) were positive for *B. pilosicoli* by qPCR (1.65×10^4^ copies/g of feces) and its identity was confirmed by sequencing. Neither *B. hyodysenteriae* nor *B. pilosicoli* were detected in any other pigs.

## Discussion

Swine dysentery, which by definition is caused by *B. hyodysenteriae* [17] ¸ has recently undergone a period of relative quiescence in North America. The emergence of a clinically indistinguishable illness associated with a distinct organism, *“B. hampsonii”* strain 30446 poses diagnostic challenges for producers, veterinarians and diagnosticians [6], [7], [18], [19]. The lack of pathognomonic clinical or pathological findings associated with *“B. hampsonii”* strain 30446 necessitates a reliance on laboratory tests to make a specific etiological diagnosis. Unfortunately there is no standardized method of identification. Although there have been no attempts to standardize testing amongst laboratories to date, the use of direct examination, culture followed by genus specific PCR and sequencing, and species-specific PCR are recommended. A number of *Brachyspira* species specific PCR assays have been published [19]–[22] but due to the apparent widespread use of techniques developed in house or modifications to published protocols, the relative sensitivity and specificity of these assays are unknown. Thus, when using PCR, negative results should be interpreted with caution. In light of these challenges, evaluation of fecal smears may be invaluable diagnostically. While fecal smears cannot differentiate between *Brachyspira* species, the presence or absence of spirochetes is useful for interpreting molecular test results.

This research confirms that *“Brachyspira hampsonii”* strain 30446 induces a mucohaemorrhagic diarrhea and colitis in pigs that is indistinguishable from swine dysentery. Trial 1 preceded our ability to grow *“B. hampsonii”* strain 30446 in broth culture, but the development of JBS broth in October 2011 made it possible to use a pure culture inoculum in trial 2. This pure broth culture was prepared from the tissue inoculum used in trial 1. The results of trial 2 demonstrate that strain 30446 causes mucohaemorrhagic diarrhea indistinguishable from the field cases where it was first observed, establishing this organism as a pathogen of swine.

The design of these trials was based on a successful, previously published trial utilizing *B. murdochii* [5]. Because the minimum infectious dose of strain 30446 is unknown, average doses of 1.95×10^8^ genome equivalents in trial 1 and 3.19×10^8^ genome equivalents in trial 2 were used, intermediate to those used in previous studies. Disease developed between four and ten days PI in trial 1 and five to nine days PI in trial 2 indicating that a dose sufficient to cause disease was used. Interestingly, the incubation period observed in these trials was consistent with an infection trial conducted as part of the initial description of swine dysentery in 1921 [1].

Pre-screening fecal samples of experimental pigs revealed a low level of *“B. hampsonii”* strain 30446 colonization (reported as DNQ) in 3/18 pigs in trial 1, and 12/18 pigs in trial 2 based on qPCR. In addition, 1 pig in trial 2 (#684) had 6.80×10^4^ genome equivalents/g of *“B. hampsonii”* strain 30446 in feces on D0 (Table 2). This finding suggests that strain 30446 is not an obligate pathogen but instead causes disease when present in sufficient numbers or when host defences are compromised. The use of strain 30446 negative pigs was preferable, but none were available at the time this research was undertaken. Low (DNQ) pre-challenge levels of *“B. hampsonii”* strain 30446 however, clearly did not induce sufficient mucosal immunity to protect against disease as evidenced by the 3/3 trial 1 pigs and 6/8 trial 2 pigs that developed mucohaemorrhagic diarrhea following challenge. Noteworthy is pig #684 that did not develop mucohaemorrhagic diarrhea following challenge, suggesting that shedding ∼10^4^ genome equivalents/g feces may have been associated with sufficient mucosal immunity to protect against disease. The results of a number of diagnostic cases completed by our team demonstrates a similar trend whereby clinical cases typically have greater than 10^5^ *“B. hampsonii”* strain 30446 genome equivalents/g of tissue or feces, whereas age-matched non-clinical animals in the same airspace have fewer than 10^5^ genomic copies/g [23]. Pre-trial colonization of CTRL animals, all of which remained healthy, supports the conclusion that DNQ levels of strain 30446 in feces are incidental. These findings are consistent with a previous report that concluded that concentrations of *B. hyodysenteriae* greater than 10^5^ CFU/gram of feces are required for the development of lesions [24].

Three pigs in trial 1 and four pigs in trial 2 did not develop mucohaemorrhagic diarrhea in spite of being inoculated. In trial 2, one pig (#683) only received two of three inoculum doses, and one (#684) was the pig with 10^4^ copies/g strain 30446 on D0. Whether failure to produce disease in these seven animals reflects normal biological variation, pre-existing immunity or the use of a marginally infectious dose is unknown.

Pathological findings in affected pigs, characterized by mucoid or mucohaemorrhagic colitis and muco-fibrinous typhlitis, were consistent with previous reports describing swine dysentery [25]. Histopathological findings in the caecum and colon of affected pigs were consistent with, but mild in comparison to gross lesions. The mild thickening and congestion seen in the ileum of four INOC pigs (one unaffected and three with mucohaemorrhagic diarrhea) in trial 2, mimicked early or mild proliferative ileitis caused by *L. intracellularis,* however the ileum of all pigs were negative by PCR for *L. intracellularis* and in no pigs were lesions typical of ileitis seen histologically. Whether or not these mild ileal lesions are a feature of strain 30446 associated disease or an incidental finding is unknown.

A number of atypical or novel, phylogenetically distinct, strongly β-haemolytic *Brachyspira* have been reported to cause disease in pigs [19]. Based on partial *nox* sequence, strain 30446 clusters separately from the other known species with good bootstrap support (Figure 1). The partial *nox* sequence for strain 30446 is identical to the provisionally named *“B. hampsonii”*, and 94.8% similar over 810 bp to *Serpulina* sp. P280/1, a porcine clinical isolate from the United Kingdom [9], [26], [27]. It is approximately 92% identical to the *B. innocens,* and *B. murdochii* strains examined, which is less similar than these two species are to each other (pairwise identities between *B. innocens* and *murdochii* are 96–97% over this same region). Furthermore, three of seven primer sets from a previously published MLST scheme for *Brachyspira* sp, failed to yield a product, while novel sequences were generated for the other four.

Two recent reports describe infection experiments using murine [28] and porcine [19] models of swine dysentery with North American *Brachyspira* strains including *“B. hampsonii”* strain 30446 isolated from pigs with signs of swine dysentery in Iowa between 2008 and 2011. These experiments were the first to demonstrate the causal relationship between strain 30446 and mucohaemorrhagic typhlocolitis in pigs. The authors found that strongly β-haemolytic strains of *Brachyspira* spp., including strain 30446, produced disease and colonic lesions typical of those associated with *B. hyodysenteriae.* In the porcine experiment [19], 4 of 10 pigs inoculated with strain 30446 had diarrhea on days 7 and 14 post infection, and 2 of 10 were culture or PCR positive at necropsy on day 16. By contrast, the incidence of mucohaemorrhagic diarrhea reported here in trial 1 and 2 was 75% and 67% respectively in INOC pigs. Furthermore, following inoculation with pure broth culture, strain 30446 was isolated by culture from 10/12 pigs, was detected by PCR in feces in 11/12 pigs and repeatedly for 3 or more days in 8 pigs. Testing for other relevant pathogens including PRRS virus, *Lawsonia intracellularis and Salmonella* spp., were negative. Collectively, these data provide substantive evidence of causality and provide the first report characterizing fecal shedding using a Canadian *“B. hampsonii”* 30446 isolate. While strain 30446 used for the present research clearly falls within *“B. hampsonii”* clade 2, the relationship between this strain and other members of the species, particularly clade 1, is unclear. Hence, additional research is necessary to more fully understand the clinical relevance of this novel and diverse *Brachyspira* species in swine.

In summary, our results confirm the causal relationship between *“Brachyspira hampsonii”* strain 30446 and mucohaemorrhagic diarrhea in swine. The emergence of *“Brachyspira hampsonii”* strain 30446 therefore poses potential diagnostic challenges since the specificity of some currently used PCR assays may not detect this organism, and *Brachyspira* culture is not widely used in diagnostic laboratories in many countries. To date, cases have been diagnosed in pigs from Alberta, Saskatchewan, Iowa, Illinois, Minnesota, Missouri and North Carolina [7], [9]. The prevalence of carrier animals, risk factors for infection, non-porcine reservoirs, antimicrobial susceptibility, minimal infectious dose and the efficacy of cleaning practices for eliminating *“B. hampsonii”* strain 30446 are entirely unknown. Research is ongoing in our lab to address these and other important questions.
